# A Large Cardiac Mass: Diagnosis of Caseous Mitral Annular Calcification and Determining Optimum Management Strategy

**DOI:** 10.1155/2014/637374

**Published:** 2014-06-17

**Authors:** Emanuel A. Shapera, Afshin Karimi, Luis R. Castellanos

**Affiliations:** ^1^UCSD School of Medicine, La Jolla, CA, USA; ^2^Department of Radiology, UCSD School of Medicine, La Jolla, CA, USA; ^3^Department of Medicine and Division of Cardiovascular Medicine, UCSD Sulpizio Family Cardiovascular Center, La Jolla, CA 92093, USA

## Abstract

A 64-year-old woman with dizziness and blurry vision underwent an evaluation for a possible stroke with a head-neck CT scan and a transthoracic echocardiogram. The head-neck CT scan was unremarkable, but the echocardiogram was notable for a 2.0 × 2.3 cm heterogeneous echodensity attached to the mitral valve. After a transesophageal echocardiogram and chest CT scan, the mass was determined to be a caseous mitral annular calcification, CMAC. This entity is a rare variant of MAC with an estimated prevalence of 0.068%. Echocardiographic techniques can distinguish CMAC from other intracardiac masses such as tumor, cyst, or abscess. CMAC is associated with cerebrovascular accidents; however, optimal treatment is controversial given the rarity of this clinical finding. Management strategies should be tailored based on the patient's presentation, risk factors, and overall clinical circumstances.

## 1. Introduction

Mitral annular calcification (MAC) describes a condition in which the annulus of the mitral valve becomes calcified [1–3]. In rare instances, these masses can have a necrotic core and are referred to as caseous mitral annular calcification (CMAC). Risk factors for developing CMAC are similar to those for developing atherosclerosis [1]. Furthermore, patients with CMAC are more likely to experience cerebrovascular accidents than the general population. The diagnosis of CMAC can be challenging, and the treatment is controversial. Anticoagulation with warfarin or direct thrombin inhibitors have been used, but, in some instances, surgical resection is warranted. In this report, we discuss the challenges that are encountered when treating a patient newly diagnosed with CMAC.

## 2. Case Report 

**Figure 1:**
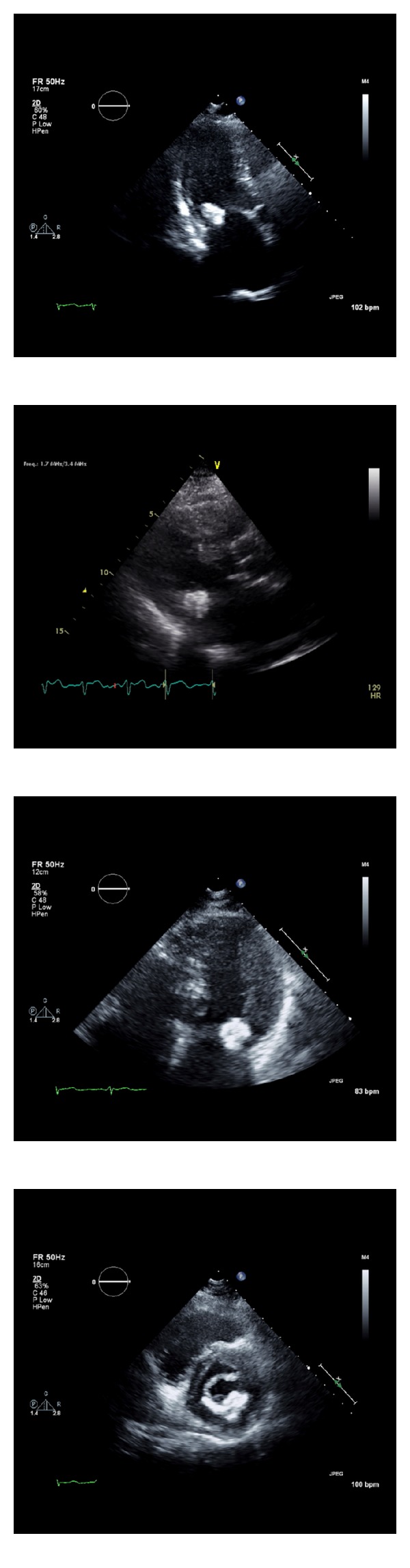
Transthoracic echocardiogram views. Four standard views that show a round echodense mass measuring 20 × 23 mm along the mitral annulus and attached to the posterior mitral leaflet.

**Figure 2:**
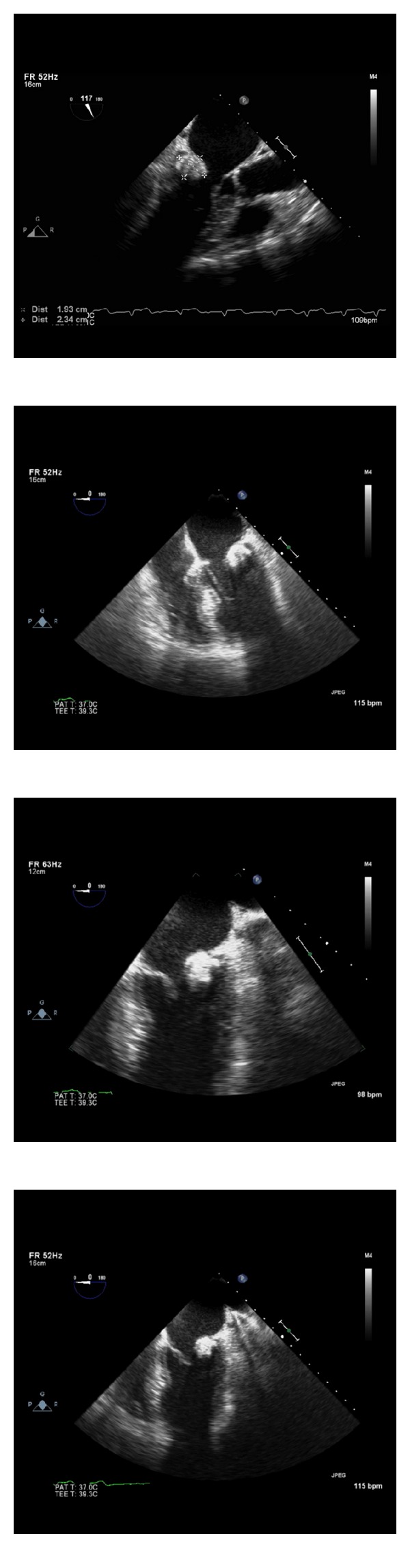
Transesophageal echocardiogram views. Four standard views that show a well-circumscribed echogenic mass that measures 19 × 23 mm and appears to be attached to mitral annulus and extending into the posterior mitral valve leaflet.

**Figure 3:**
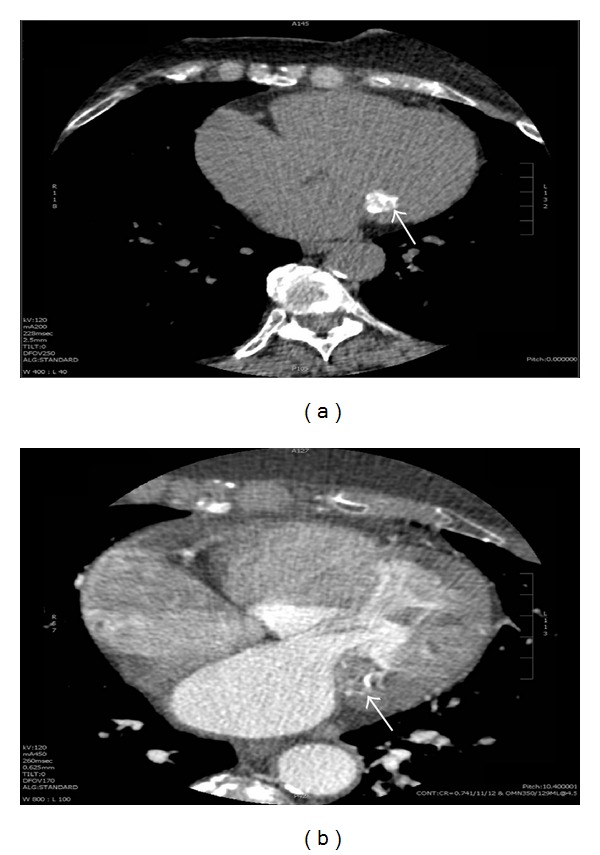
Chest CT scan without and with contrast. A 17 × 18 mm round hyperdense mass was identified (white arrow) along the mitral valve annulus (a). There is a heterogeneous calcification pattern with a hypoattenuated necrotic center (b).

A 64-year-old woman was admitted to the hospital after experiencing acute visual deficits. Her symptoms resolved within 24 hours and were consistent with a transient ischemic attack (TIA). The patient's past medical history was significant for paroxysmal atrial fibrillation, migraines, and hypertension. Prior medical records indicated that she was taking 325 mg of aspirin once a day for paroxysmal atrial fibrillation. The patient did not take any additional medications. Her family and social history were unremarkable. On physical exam, her blood pressure was 170/116 mmHg and heart rate was 88 bpm with a respiratory rate of 20 and oxygen saturation of 98%. The patient had an irregularly irregular heart rate and rhythm with a soft midpeaking systolic murmur best appreciated at the right upper sternal border. No carotid bruits were appreciated. An electrocardiogram showed atrial fibrillation with a ventricular rate of 88 bpm and no significant ST or T wave changes. Remarkable lab values included a prothrombin time of 11.4 s, 0.5 mg/dL creatinine, 71 mg/dL HDL, 105 mg/dL LDL, and 103 mg/dL triglycerides. CT angiography of the head and neck was unremarkable except for mild carotid bulb calcifications. Brain MRI revealed old ischemic changes in the periventricular and subcortical white matter consistent with possible old strokes. A transthoracic echocardiogram (TTE) noted a round echodense mass measuring 20 × 23 mm attached to the mitral valve annulus, along the atrioventricular groove and adjacent to the posterior mitral leaflet (Figure 1). The mass was described as having a smooth border, possibly tumor versus thrombus. There was mild mitral valve thickening with mild mitral regurgitation, but no stenosis or LV outflow tract obstruction. There was aortic valve sclerosis without stenosis. The left ventricle had a normal size with a preserved ejection fraction (69% LVEF biplane) and no wall motion abnormalities. Given the abnormal valvular findings, a transesophageal echocardiogram (TEE) was recommended. It revealed a well-circumscribed echogenic and nonmobile mass attached to the posterior mitral valve annulus measuring 19 × 23 mm (Figure 2). The subvalvular mitral apparatus was intact and the left atrial appendage did not show a thrombus. Based on the size, shape, and location of the mass, the differential diagnosis included myxoma versus caseous mitral annular calcification. A nongated contrast-enhanced chest CT scan showed a 17 × 18 mm round hyperdense mass along the inferior mitral valve annulus (Figure 3). The mass was described as having a heterogenous calcification pattern with a hypoattenuated necrotic center of 100 Hounsfield units. These findings were consistent with caseous mitral annular calcification. In light of the newly identified ischemic changes on her brain MRI and most recent CVA episode, there was concern for possible embolization from the CMAC complex. The patient was evaluated by cardiothoracic surgery and was deemed an operable candidate, but the patient declined surgery. Given these findings and a calculated CHADS_2_ score of 3, the patient was started on rivaroxaban, a factor Xa inhibitor for stroke management. She was followed closely with a repeat TTE 6 months later revealing no changes in the size or appearance of the mass.

## 3. Discussion 

Caseous mitral annular calcification is a rare medical condition that involves the mitral annulus and, in some instances, extends into the mitral valve. Deluca et al. [2] found that 2,169 out of 20,468 patients (10.6%) who had been referred for a routine echocardiogram were found to have MAC. Of the 2,169 patients, 14 (0.64%) had CMAC [2]. A similar study by Harpaz et al. evaluated 28,364 patients with TTE and found 19 to have CMAC (0.63%) [3]. These two studies estimated the prevalence of CMAC to be 0.067% in all patients referred for a TTE.

The risk factors for developing CMAC are similar to those for developing atherosclerosis [4]. Since age is a risk factor in the development of atherosclerosis, the prevalence of CMAC also rises with age [5]. Pomerance showed in one necropsy series involving 258 cases of MAC that the prevalence of CMAC in persons over the age of 50 was 2.7% in comparison to a prevalence of 0.67% in the entire series [5].

Cardiac masses are uncommon findings that can involve thrombus, malignant or benign tumors, metastases, and vegetations. Nonmalignant tumors such as myxomas can have a characteristic stalk attaching the mass to the interatrial septum [6]. CMAC produce characteristic findings on imaging that reflects their composition. Imaging identifies these masses as having a dense rim surrounding a more lucent necrotic center [7]. Although TTE can be useful due to its less invasive nature, TEE can better classify the number, location, and character of intracardiac masses [8], thus potentially altering the management of the patient. CT can be applied to illuminate calcifications with central areas of noncalcified material such as a necrotic core. It has been reported by Blankstein et al. that the surrounding rim was 300 Hounsfield units whilst the necrotic core was 70 Hounsfield units [9]. The unique character of this mass on imaging has been discussed in the literature as an adjunct in assisting physicians in making the correct diagnosis and potentially avoiding unnecessary surgical interventions [10].

There does not appear to be a consensus as to what constitutes the best management for patients diagnosed with CMAC. Some studies have shown that patients with CMAC have an increased risk of developing atrial fibrillation when compared to patients without CMAC after controlling all other cerebral and cardiovascular risk factors [11]. Harpaz et al. was able to demonstrate that for every millimeter increase in the width of a CMAC mass, there was an increase in stroke risk by a relative risk ratio of 1.24 (95% CI 1.12–1.37) [3]. Findings from the Framingham cohort study showed that after controlling for traditional cardiac risk factors, patients with MAC were twice as likely to experience a stroke than patients without MAC [12]. There is no clear consensus for a mechanism to account for this association between CMAC and stroke. Plausible explanations for the association between CMAC and stroke include an embolism of a fragment of the CMAC complex as well as changes in cardiac structure from the heavily calcified annulus, thus causing left atrium enlargement and subsequent rhythm disturbances that predisposes the patient to cardiac thrombus formation.

This rare clinical case illustrates how echocardiography and allied imaging modalities can help differentiate CMAC from other intracardiac masses. Making the correct diagnosis of CMAC can facilitate the implementation of appropriate treatment strategies that may help reduce the risk of future cerebrovascular accidents.

## References

[1] Benjamin EJ, Plehn JF, D’Agostino RB (1992). Mitral annular calcification and the risk of stroke in an elderly cohort. *The New England Journal of Medicine*.

[2] Deluca G, Correale M, Ieva R, Salvatore BD, Gramenzi S, Di Biase M (2008). The incidence and clinical course of caseous calcification of the mitral annulus: a prospective echocardiographic study. *Journal of the American Society of Echocardiography*.

[3] Harpaz D, Auerbach I, Vered Z, Motro M, Tobar A, Rosenblatt S (2001). Caseous calcification of the mitral annulus: a neglected, unrecognized diagnosis. *Journal of the American Society of Echocardiography*.

[4] Adler Y, Herz I, Vaturi M (1998). Mitral annular calcification detected by transthoracic echocardiography is a marker for high prevalence and severity of coronary artery disease in patients undergoing coronary angiography. *The American Journal of Cardiology*.

[5] Pomerance A (1970). Pathological and clinical study of calcification of the mitral valve ring. *Journal of Clinical Pathology*.

[6] Obeid AI, Marvasti M, Parker F, Rosenberg J (1989). Comparison of transthoracic and transesophageal echocardiography in diagnosis of left atrial myxoma. *The American Journal of Cardiology*.

[7] Ribeiro S, Salgado A, Salomé N (2012). Caseous calcification of the mitral annulus: a multi-modality imaging perspective. *Revista Portuguesa de Cardiologia*.

[8] Mugge A, Daniel WG, Haverich A, Lichtlen PR (1991). Diagnosis of noninfective cardiac mass lesions by two-dimensional echocardiography. Comparison of the transthoracic and transesophageal approaches. *Circulation*.

[9] Blankstein R, Durst R, Picard MH, Cury RC (2009). Progression of mitral annulus calcification to caseous necrosis of the mitral valve: complementary role of multi-modality imaging. *European Heart Journal*.

[10] Pomeroy WL, Markelz B, Steel K (2013). Mitral annular caseous calcification: a rare variant of a common echocardiographic finding discovered with advanced imaging techniques. *Case Reports in Medicine*.

[11] Aronow WS, Koenigsberg M, Kronzon I, Gutstein H (1990). Association of mitral anular calcium with new thromboembolic stroke and cardiac events at 39-month follow-up in elderly patients. *The American Journal of Cardiology*.

[12] Fox CS, Vasan RS, Parise H (2003). Mitral annular calcification predicts cardiovascular morbidity and mortality: the Framingham Heart Study. *Circulation*.

